# Identification of variants in genes associated with hypertrophic cardiomyopathy in Mexican patients

**DOI:** 10.1007/s00438-023-02048-8

**Published:** 2023-07-27

**Authors:** Catalina García-Vielma, Luis Gerardo Lazalde-Córdova, José Cruz Arzola-Hernández, Erick Noel González-Aceves, Herminio López-Zertuche, Nancy Elena Guzmán-Delgado, Francisco González-Salazar

**Affiliations:** 1 Centro de Investigación Biomédica del Noreste, Departamento de Citogenética, Instituto Mexicano del Seguro Social, Monterrey, NL México; 2 Departamento de Electrofisiología, Instituto Mexicano del Seguro Social, Unidad Médica de Alta Especialidad. Hospital de cardiología No. 34 “Dr. Alfonso J. Treviño Treviño” del Centro Médico Nacional del Noreste, Monterrey, NL México; 3 Instituto Mexicano del Seguro Social, Hospital General de Zona No. 4, Monterrey, NL México

**Keywords:** Hypertrophic cardiomyopathy, Sudden death, *MYH7*, *MYBPC3*, Gene variants

## Abstract

The objective of this work was to identify genetic variants in Mexican patients diagnosed with hypertrophic cardiomyopathy (HCM). According to world literature, the genes mainly involved are MHY7 and MYBPC3, although variants have been found in more than 50 genes related to heart disease and sudden death, and to our knowledge there are no studies in the Mexican population. These variants are reported and classified in the ClinVar (PubMed) database and only some of them are recognized in the Online Mendelian Information in Men (OMIM). The present study included 37 patients, with 14 sporadic cases and 6 familial cases, with a total of 21 index cases. Next-generation sequencing was performed on a predesigned panel of 168 genes associated with heart disease and sudden death. The sequencing analysis revealed twelve (57%) pathogenic or probably pathogenic variants, 9 of them were familial cases, managing to identify pathogenic variants in relatives without symptoms of the disease. At the molecular level, nine of the 12 variants (75%) were single nucleotide changes, 2 (17%) deletions, and 1 (8%) splice site alteration. The genes involved were MYH7 (25%), MYBPC3 (25%) and ACADVL, KCNE1, TNNI3, TPM1, SLC22A5, TNNT2 (8%). In conclusion; we found five variants that were not previously reported in public databases. It is important to follow up on the reclassification of variants, especially those of uncertain significance in patients with symptoms of the condition. All patients included in the study and their relatives received family genetic counseling.

## Introduction

Primary hypertrophic cardiomyopathy (HCM) is considered a priority health problem in Mexico (INEGI 2020; Gobierno de México 2022) and globally (Antzelevitch 2007; Cheng et al. 2021), which in some cases begins with sudden death. Approximately 50% of HCM cases are caused by variations in genes that code for sarcomere proteins (Marian and Roberts 1995; Kimura et al. 1997; McKenna and Monserrat Iglesias 2000). More than 8000 gene variants have been identified in more than 50 genes associated with heart disease and sudden death (Coppini et al. 2014; Herrera-Rodriguez et al. 2020), most of them reported in the ClinVar database of the National Center of Biotechnology Information of PubMed (Sayers et al. 2021), and to the best of our knowledge, there have been no reports in the Mexican population.

Most cases are inherited in an autosomal dominant manner, which is why they affect both sexes equally, reaching genealogies with repetition of the disease, with incomplete penetrance and variable expressivity (Antzelevitch 2007; Maron et al. 2022). In familial cases, there are only 25 recognized variants in the Online Mendelian Inheritance in Man (OMIM) (Amberger et al. 2015; Herrera-Rodriguez et al. 2020) and less than 5% of cases have more than one variant with the severity of the phenotype due to gene dose effect (Wang et al. 2014; Rafael et al. 2017).

The genes most frequently associated with HCM are *MYH7* and *MYBPC3*, in 15–25% of cases. Others such as *TNNT2* and *TNNI3* are found with frequencies lower than 5% (Ross et al. 2017; Herrera-Rodriguez et al. 2020). The types of gene variants that can be found are pathogenic and probably pathogenic (*PV, PPV*) variants that increase or probably increase the predisposition to the disease, benign or probably benign (*BV, PBV*) variants, which are not associated with the disease, and variants of uncertain significance (*VUS*), in which it is unknown whether or not it can contribute to the development of the disease (Alyousfi et al. 2021; Sayers et al. 2021; Richards et al. 2015).

The objective of this study is to identify genetic variants in Mexican patients with a previous clinical diagnosis of HCM through next-generation sequencing (NGS) with a panel of 168 genes associated with heart disease and sudden death.

## Material and methods

### Patients

Male and female patients, of any age, with a previous diagnosis of HCM were recruited. The diagnosis was established by specialists in cardiology from the Mexican Institute of Social Security, based on the guidelines of the American College of Cardiology (Ommen et al. 2020). All patients agreed to participate in the study and signed a written informed consent; in minors, the consent was signed by one of their parents. In patients with a positive molecular result for any PV or PPV, their relatives were invited to participate, exploring their family history with suspected HCM or sudden death in the family, before 60 years of age. Patients who reported a family history were taken as family cases and patients without a family history were considered sporadic.

### Genetic study

The DNA was extracted from a peripheral blood sample of the patients. Subsequently, NGS (Rubio et al. 2020) using a hybridization-based protocol, and sequenced using Illumina technology was performed with a predesigned genetic panel named Invitae Arrhythmia and Cardiomyopathy Comprehensive panel, of 168 genes associated with cardiomyopathies and sudden death (Table 1). These genes were selected using oligonucleotide primers designed to capture exons, the 10–20 bases flanking intronic sequences, and certain noncoding regions of interest (Agilent Technologies, Santa Clara, CA; Roche, Pleasanton, CA; Integrated DNA Technologies, Coralville, AI). The selected gene regions were sequenced with an average coverage of 350⨉ (50 ⨉ minimum). The GRCh37 reference genome database was used for single nucleotide variants (SNVs), small and large insertions/deletions (indels), structural variants, and intragenic copy number variants (Truty et al. 2019). Clinically significant variants not meeting strict NGS quality metrics were confirmed using an orthogonal method (Lincoln et al. 2019). Enrichment and analysis focus on the coding sequence of the indicated transcripts, 20 bp of flanking intronic sequence, and other specific genomic regions demonstrated to be causative of disease at the time of assay design. Markers across the X and Y chromosomes are analyzed for quality control purposes and may detect deviations from the expected sex chromosome complement. Detected variants were interpreted using Sherloc (semiquantitative, hierarchical evidence-based rules for locus interpretation), (Nykamp et al. 2017), using a point-based system incorporating the American College of Medical Genetics and Association of Molecular Pathology (ACMG–AMP) joint consensus statement guidelines (Ommen et al. 2020; Richards et al. 2015) and classified as: PV, PPV, BV, PBV, and VUS (den Dunnen and Antonarakis 2014; Richards 2015). Rare variants were defined as those with a minor allelic filtering frequency [MAF] < 1.0e − 4 based on a public data set.

**Table 1:** Genes sequenced in patients with HCM

*ABCC9:* ATP binding cassette subfamily C member 9	*ACADVL:* Acyl-CoA dehydrogenase very long chain	*ACTC1:* Actin, Alpha, Cardiac muscle
*ACTN2:* ACTININ, ALPHA-2	*ADNJC19:* DnaJ Heat Shock Protein Family (Hsp40) Member C19	*AGL:* Amylo-1,6-Glucosidase, 4-Alpha-Glucanotransferase
*AKAP9:* A-kinase anchoring protein 9	*ANK2:* Ankyrin 2	*ANKRD1:* Ankyrin Repeat Domain 1
*ALMS1:* Centrosome And Basal Body Associated Protein	*ALPK3:* Alpha Kinase 3	*A2ML1:* Alpha-2-macroglobulin like 1
*BAG3:* Bcl2-Associated Athanogene 3	*BRAF:* B-Raf Proto-Oncogene, Serine/Threonine Kinase	*CACNA1C:* Calcium Voltage-Gated Channel Subunit Alpha1 C
*CACNA1D:* Calcium Voltage-Gated Channel Subunit Alpha1 D	*CALM1:* Calmodulin 1	*CALM2*: Calmodulin 2
*CACNA2D1:* Calcium Voltage-Gated Channel Auxiliary Subunit Alpha2delta 1	*CACNB2:* Calcium Voltage-Gated Channel Auxiliary Subunit Beta 2	*CALR3:* Calreticulin 3
*CALM3:* Calmodulin 3	*CASQ2:* Calsequestrin 2	*CBL:* Cbl proto-oncogene
*CAV3:* Caveolin 3	*CHRM2:* Cholinergic Receptor Muscarinic 2	*CTF1:* Cardiotrophin 1
*CRYAB:* Crystallin Alpha B	*CSRP3:* Cysteine and Glycine Rich Protein 3	*DTNA:* Dystrobrevin Alpha
*CTNNA3:* Catenin Alpha 3	*CDH2:* Cadherin 2	*CPT2:* Carnitine Palmitoyltransferase 2
*DEPDC5:* DEP domain containing 5, GATOR1 subcomplex subunit	*ELAC2:* Elac Ribonuclease Z 2	*KCNA1:* Potassium Voltage-Gated Channel Subfamily A Member 1
*DES:* Desmin	*DMD:* Dystrophin	*DOLK:* Dolichol Kinase
*DSC2:* Desmocollin 2	*DSG2:* Desmoglein 2	*DSP:* Desmoplakin
*EYA4:* EYA Transcriptional Coactivator And Phosphatase 4	*FHL1:* Four-and-a-half LIM domains 1	*FHL2:* Four-and-a-half LIM domains 2
*FKRP:* Fukutin Related Protein	*FKTN:* Fukutin	*FLNC:* filamin C
*GAA:* Alpha glucosidase	*GATA4:* GATA Binding Protein 4	*GATA5:* GATA Binding Protein 5
*GATA6:* GATA Binding Protein 6	*GATAD1:* GATA Zinc Finger Domain Containing 1	*GPD1L:* Glycerol-3-Phosphate Dehydrogenase 1 Like
*GJA5:* Gap Junction Protein Alpha 5	*GLA:* Galactosidase Alpha	*HAND1:* Heart And Neural Crest Derivatives Expressed 1
*HCN4:* Hyperpolarization Activated Cyclic Nucleotide Gated Potassium Channel 4	*HRAS:* HRas Proto-Oncogene, GTPase	*ILK:* Integrin Linked Kinase
*JPH2:* Junctophilin 2	*JUP:* Junction Plakoglobin	*KCNE1:* Potassium voltage-gated channel subfamily E regulatory subunit 1
*KCNE2:* Potassium Voltage-Gated Channel Subfamily E Regulatory Subunit 2	*KCNE3:* Potassium Voltage-Gated Channel Subfamily E Regulatory Subunit 3	*KCNE5:* Potassium Voltage-Gated Channel Subfamily E Regulatory Subunit 5
*KCNH2:* Potassium Voltage-Gated Channel Subfamily H Member 2	*KCNJ2:* Potassium Inwardly Rectifying Channel Subfamily J Member 2	*KCNQ1:* Potassium Voltage-Gated Channel Subfamily Q Member 1
*KCNJ5:* Potassium Inwardly Rectifying Channel Subfamily J Member 5	*KCNJ8:* Potassium Inwardly Rectifying Channel Subfamily J Member 8	*KCNK3:* Potassium Two Pore Domain Channel Subfamily K Member 3
*KCNQ2:* Potassium Voltage-Gated Channel Subfamily Q Member 2	*KCNQ3:* Potassium Voltage-Gated Channel Subfamily Q Member 3	*KCNT1:* Potassium Sodium-Activated Channel Subfamily T Member 1
*KIF20A:* Kinesin Family Member 20A	*KLF10:* KLF Transcription Factor 10	*LAMP2:* Lysosomal Associated Membrane Protein 2
*KRAS:* KRAS Proto-Oncogene, GTPase	*KCNA5*: Potassium Voltage-Gated Channel Subfamily A Member 5	*KCND3:* Potassium Voltage-Gated Channel Subfamily D Member 3
*LDB3:* LIM Domain Binding 3	*LRRC10:* Leucine Rich Repeat Containing 10	*MAP2K1:* Mitogen-Activated Protein Kinase Kinase 1
*LMNA:* Lamin A/C	*LZTR1*: Leucine Zipper Like Transcription Regulator 1	*LAMA4:* Laminin Subunit Alpha 4
*MAP2K2:* Mitogen-Activated Protein Kinase Kinase 2	*MRAS:* Muscle RAS Oncogene Homolog	*MTO1:* Mitochondrial TRNA Translation Optimization 1
*MAP3K8:* Mitogen-Activated Protein Kinase Kinase Kinase 8	*MED12:* Mediator Complex Subunit 12	*MYH6:* Myosin Heavy Chain 6
*MYBPC3:* Myosin Binding Protein C3	*MYH7:* Myosin Heavy Chain 7	*MYL2:* Myosin Light Chain 2
*MYL3:* Myosin Light Chain 3	*MYL4:* Myosin Light Chain 4	*MYLK3:* Myosin Light Chain Kinase 3
*MYLK2:* Myosin Light Chain Kinase 2	*MYOM1:* Myomesin 1	*MYOZ2:* Myozenin 2
*MYPN:* Myopalladin	*NEBL:* Nebulette	*NF1:* Neurofibromin 1
*NKX2-5:* NK2 Homeobox 5	*NRAS:* NRAS Proto-Oncogene, GTPase	*NEXN:* Nexilin F-Actin Binding Protein
*NPPA:* Natriuretic Peptide A	*PDLIM3:* PDZ And LIM Domain 3	*PLEKHM2:* Pleckstrin Homology And RUN Domain Containing M2
*PCDH19: Protocadherin 19*	*PRRT2:* Proline-Rich Transmembrane Protein 2	*SCN1A:* Sodium Voltage-Gated Channel, Alpha Subunit 1
*PLN:* Phospholamban	*PPA2:* Inorganic Pyrophosphatase 2	*PPCS:* Phosphopantothenoylcysteine Synthetase
*PPP1CB:* Protein Phosphatase 1 Catalytic Subunit Beta	*RANGRF:* RAN guanine nucleotide reléase factor	*RASA2:* RAS P21 Protein Activator 2
*PRDM16:* PR/SET Domain 16	*PCCA:* Propionyl-CoA Carboxylase Subunit Alpha	*PCCB:* Propionyl-CoA Carboxylase Subunit Beta
*PRKAG2:* Protein Kinase AMP-Activated Non-Catalytic Subunit Gamma 2	*PTPN11:* Protein Tyrosine Phosphatase Non-Receptor Type 11	*PKP2:* Plakophilin 2
*RBM20:* RNA Binding Motif Protein 20	*RIT1:* Ras Like Without CAAX 1	*RYR2:* Ryanodine Receptor 2
*RRAS:* RAS Related	*RAF1:* Raf-1 Proto-Oncogene, Serine/Threonine Kinase	*RASA1:* RAS P21 Protein Activator 1
*SCN10A:* Sodium Voltage-Gated Channel Alpha Subunit 10	*SCN1B:* Sodium Voltage-Gated Channel Beta Subunit 1	*SCN5A:* Sodium Voltage-Gated Channel Alpha Subunit 5
*SCN4B:* Sodium Voltage-Gated Channel Beta Subunit 4	*SLMAP:* Sarcolemma Associated Protein	*SNTA1:* Syntrophin Alpha 1
*SCN8A:* Sodium Voltage-Gated Channel, Alpha Subunit 8	*SCN9A:* Sodium Voltage-Gated Channel, Alpha Subunit 9	*EMD:* Emerin
*SDHA:* Succinate Dehydrogenase Complex Flavoprotein Subunit A	*SGCD:* Sarcoglycan Delta	*SHOC2:* SHOC2 Leucine Rich Repeat Scaffold Protein
*SLC22A5:* Solute Carrier Family 22 Member 5	*SOS1:* SOS Ras/Rac Guanine Nucleotide Exchange Factor 1	*SOS2:* SOS Ras/Rho Guanine Nucleotide Exchange Factor 2
*SLC2A1:* Solute Carrier Family 2 Member 1	*TAZ:* Tafazzin, Phospholipid-Lysophospholipid Transacylase	*TBX20: T*-Box Transcription Factor 20
*SPRED1:* Sprouty Related EVH1 Domain Containing 1	*SCN2B:* Sodium Voltage-Gated Channel Beta Subunit 2	*SCN3B:* Sodium Voltage-Gated Channel Beta Subunit 3
*TCAP: Titin-Cap*	*TMEM43:* Transmembrane Protein 43	*TMEM70:* Transmembrane Protein 70
*TMPO:* Thymopoietin	*TXNRD2:* Thioredoxin Reductase 2	*TTN:* Titin
*TNN13:* Troponin I3, Cardiac Type	*TNN13K:* TNNI3 Interacting Kinase	*TNNT2:* Troponin T2, Cardiac Type
*TPM1:* Tropomyosin 1	*TRDN:* Triadin	*TRPM4:* Transient Receptor Potential Cation Channel Subfamily M Member 4
*TTR:* Transthyretin	*VCL:* Vinculin	*TNNC1:* Troponin C1, Slow Skeletal And Cardiac Type

## Results

A total of 37 samples were analyzed. Of these, 14 were sporadic cases and 6 familial cases (7 index cases and 16 relatives), with a total of 21 index cases. The age range of the patients was from 7 to 83 years; 24 (65%) were women and 13 (35%) men.

Twelve (57%) 12 PV and PPV / 21 index cases, were detected in patients with an established diagnosis of HCM. Genes with gene variants were *MYH7* (25%), *MYBPC3* (25%), *ACADVL, KCNE1, TNNI3, TPM1, SLC22A5*, and *TNNT2* (1 each, 8%), of which 9 (75%) were SNVs, 2 (17%) deletions, and 1 (8%) splicing site alteration.

### Family cases

**Fig. 1:**
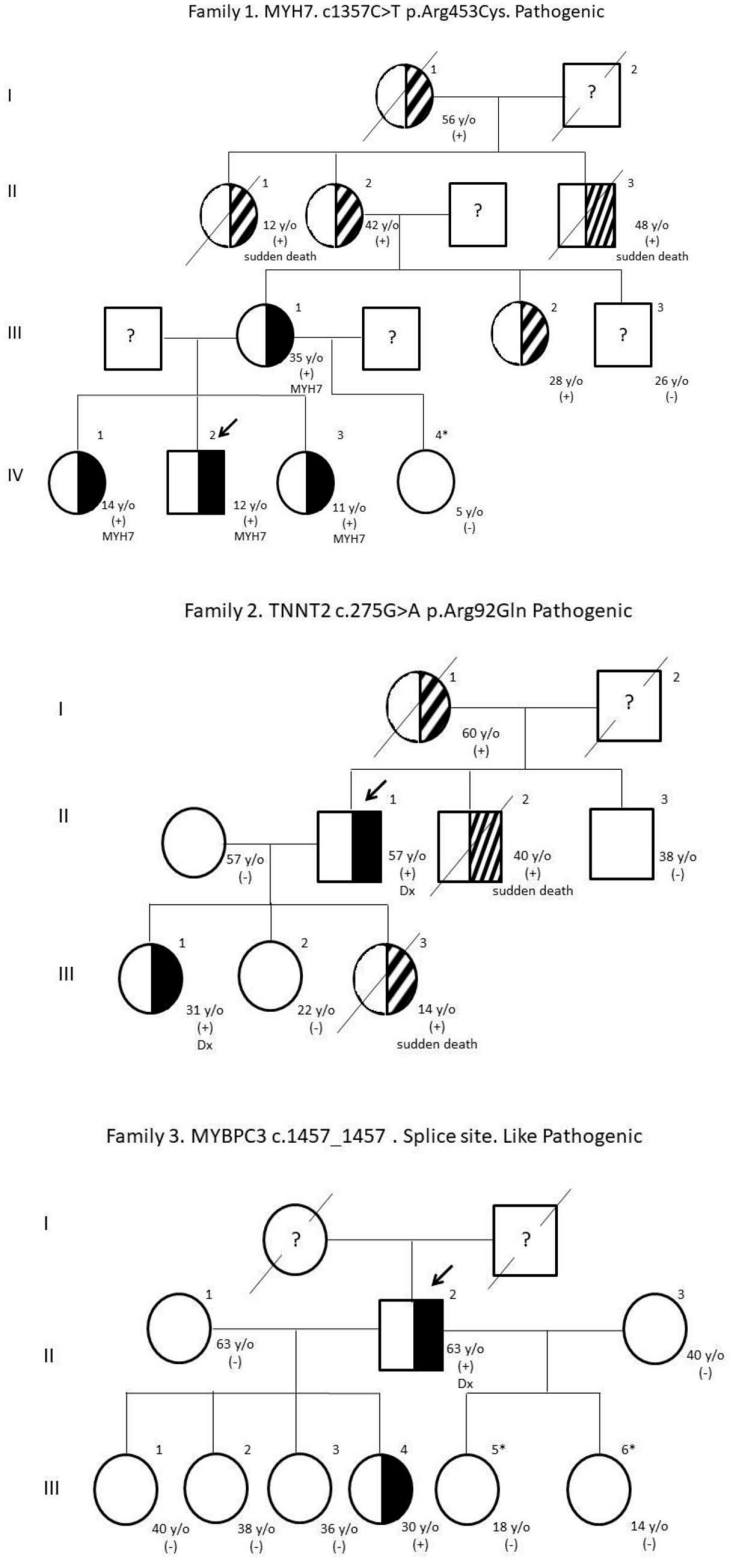

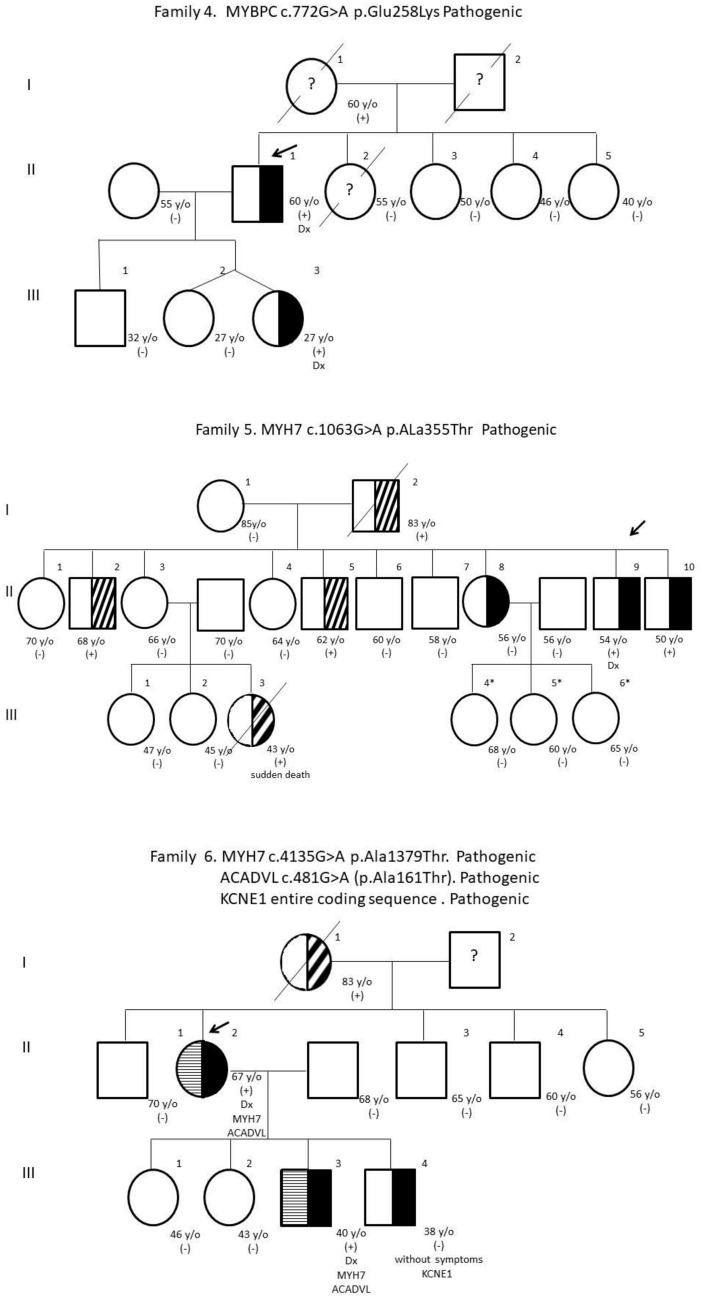
Pedigree and gene variants of the families studied

Eight PV and PPV were detected in families with a history of HCM. The genealogical trees of the families are presented (Fig. 1) and the gene variants found, indicating whether or not they are reported in the ClinVar database, their variant number, and their probable consequence at the molecular level (Table 2) (den Dunnen and Antonarakis 2014).

**Table 2:** Gene variants found in the Mexican patients studied

Family	Gene	cDNA	Aminoacid change	Variant type	ID variant ClinVar	Change type	Molecular consecuence
1	*MYH7*	*c.1357C* > *T*	*p.Arg453Cys*	PV	14,089.22	SNV	Missense
2	*TNNT2*	*c.275G* > *A*	*p.Arg92Gln*	PV	N/R	SNV	?
3	*MYBPC3*	*c.1457_1457*	*Splice site*	PPV	N/R	Splice-site variant	Loss of exons or inclusion of introns that alter the protein sequence
4	*MYBPC3*	*c.772G* > *A*	*p.Glu258Lys*	PV	42,792.35	SNV	Missense
5	*MYH7*	*c.1063G* > *A*	*p.Ala355Thr*	PV	42,820.25	SNV	Missense
6	ACADVL*	*c.481G* > *A*	*p.Ala161Thr*	PV	N/R	SNV	?
6	KCNE1*	*Deletion*	*Entire coding sequence*	PV	N/R	deletion	Absence of protein
6	*MYH7*	*c.4135G* > *A*	*p.Ala1379Thr*	PV	42,993.12	SNV	Missense
Sporadic	*MYBPC3*	*c.1800del*	p.Lys600Asnfs*2	PV	42,568.17	deletion	Stop
Sporadic	SLC22A5*	*c.695C* > *T*	*p.Thr232Met*	PV	25,386.25	SNV	Missense
Sporadic	*TNNI3*	*c.470C* > *T*	*p.Ala157Val*	PV	43,388.22	SNV	Missense
Sporadic	*TPM1*	*c.62G* > *T*	*p.Arg21Leu*	PPV	N/R	SNV	?

*SNV* single nucleotide variant, *N/R* ClinVar not reported, *PV* pathogenic variant, *PPV* like−pathogenic variant

*Gene variant not included in the OMIM

? Unknown

#### Family 1

The son (IV-2) is the index case; he has an established diagnosis of HCM, the mother (*III-1*) and the daughters (*IV-1* and *IV-3*) have symptoms. In all of them, an SNV was found in the *MYH7* gene, in a heterozygous state, with a change in codon 1357 from cytosine (*C*) to thymine (*T*), which causes a change in amino acid 453 from arginine (*Arg*) to cysteine (*Cys*), considered as PV. The other daughter (*IV-4*) has no symptoms and was negative for PV. This family has a history of two cases of sudden death (*II-1* and *II-3*).

#### Family 2

Cases *II-1* and *III-1* have an established diagnosis of HCM. They have a history of two sudden deaths in different generations (*I-2* and *III-3*). In both patients, a variant in *TNNT2* was found, in the heterozygous state, with a change in codon 275 from guanine (*G*) to alanine (*A*) and a change in amino acid 92 from *Arg* to glutamine (*Gln*), considered as PV.

#### Family 3

Patient *II-2* is the index case, with an established diagnosis of HCM. A variant was found in *MYBPC3*, with a change in codon 1457 in a splicing site cataloged as PPV. Daughter *III-4* was also heterozygous for PPV, with no clinical symptoms. Daughters *III-5* and *III-6* were also studied and were negative for PPV. There is no history of sudden death or major cardiac events in previous generations.

#### Family 4

Patient *II-1* is the index case with an established diagnosis of HCM. A PV was found in *MYBPC*, with a change at codon 772 from *G* to *A*, causing a change at amino acid 258 from glutamic acid (*Glu*) to lysine (*Lys*). Daughter *III-3* was also heterozygous for PV without having an established diagnosis of HCM. Her twin sister (*III-2*) has no symptoms and it was not possible to perform the molecular study on her. Apparently, there is no history in previous generations.

#### Family 5

This is a very large family where the index case was patient *II-9* with an established diagnosis of HCM. A PV was found in *MYH7*, in the heterozygous state, with a change in codon 1063 from *G* to *A*, which leads to a change in amino acid 355 from alanine (*Ala*) to Threonine (*Thr*). Siblings *II-8* and *II-10* were also heterozygous for PV, without presenting symptoms of the disease. The daughters of patient II-8 (*III-4, III-5,* and *III-6*) were studied and were negative for PV. This family has a history of sudden death (*III-3*) and other family members with symptoms did not agree to be analyzed.

#### Family 6

The index case in this family is patient *II-2* with an established diagnosis of HCM. This patient was double heterozygous for two PVs, the first in *MYH7* with a change in codon 4135 from *G* to *A*, which modifies amino acid 1379 from *Ala* to *Thr*, and the second in *ACADVL* with a change in codon 481 from *G* to *A*, with change in amino acid 161 of *Ala* for *Thr*. In son *III-3*, he presented symptoms of heart disease, without having an established diagnosis of HCM, and he also turned out to be double heterozygous for the same PVs in *MYH7* and *ACADVL*. Son *III-4* does not present symptoms of heart disease and was heterozygous but for a different PV located in *KCNE1*, which is caused by a deletion of the entire coding sequence, without presenting the other PV in *MYH7* and *ACADVL* that his mother and brother have. This family has a history of heart disease in case II-2.

### Sporadic cases

PV and PPV were found in 4 cases (29%) of the 14 patients with no history of HCM in the family, of which 3 PV were found in *MYBPC3, TNNI3*, and *SLC22A5* and one PPV in *TPM1* (Table 2). The rest of the sporadic cases were negative for PV and PPV.

### Genetic counselling

All HCM patients included in this study and their relatives were referred to a geneticist for genetic counselling, regardless of the type of variant found. The classification of genetic variants may change as the databases are fed back with results from new studies. It is important to monitor these variants, especially those VUS found in patients with severe symptoms of the disease.

## Discussion

PV and PPV were identified in 57% (12/21) of the patients analyzed with an established diagnosis of HCM. The genes involved are similar to those previously reported in the literature *MYH7* (25%) and *MYBPC3* (25%) (Amberger et al. 2015; Chiou et al. 2015; Herrera-Rodriguez et al. 2020) and *TNNI3, TPM1,* and *TNNT2* (8%), (García-Castro 2009; Herrera-Rodriguez et al. 2020). Of the 12 PV and PPV found, 7 are reported in ClinVar (Sayers et al. 2021) (Table 2) and the other 5 are not found in this database, but they were designated as PV and PPV by the ACMG–AMP variant classification criteria (Nykamp et al. 2017). The PVs in *ACADVL, KCNE1*, and *SLC22A5* are not included in the OMIM within the 25 most frequent variants in HCM (Amberger et al. 2015). At the molecular level, we found 9 (75%) SNVs that lead to changes in the amino acid sequence of the protein and prevent its correct functioning (Amberger et al. 2015), 2 deletions (17%), and 1 alteration in the splicing site (8%). Presence of PV and PPV in these genes makes it possible to improve the follow-up of carrier patients, offering genetic counseling to the family depending on their mode of inheritance. As well as early management of relatives who did not present symptoms of the disease.

Previous investigations in other populations report 54.2, 60.6 and 43.8% in the United States, France, and Japan, respectively (Richard et al. 2003; Van Driest et al. 2004; Otsuka et al. 2012). This percentage can be explained as our population was clinically selected and a history of severe heart disease and sudden death was considered in family cases.

Genes with variants encode or are associated with sarcomeric proteins and the change found causes that had some effect, or absence of protein formation, or they are related to ion transport processes associated with HCM. The *MYH7* gene (OMIM 160760) is located on chromosome 14 at position q11.2 and codes for the heavy chain of β-myosin, involved in cardiac muscle contraction (Perrot et al. 2005; O’Leary et al. 2016). In families 1, 5, and 6, PVs were found in this gene, all with a single nucleotide change and previously reported (Burns et al. 2017, Nykamp et al. 2017, Salazar-Mendiguchia et al. 2020). In this gene, the gene variant that changes *Arg* to *Cys* at position 453 has been reported to have a more aggressive phenotype, due to a change in amino acid charge, compared with other reported variants (Epstein et al. 1992; Frisso et al. 2009).

The *MYBPC3* gene (OMIM 6000958) (Amberger et al. 2015) is located at 11p11.2 and codes for myosin-binding protein C. The molecular consequence of the deletion found in this gene is the formation of a premature termination codon, which results in an absent or altered protein. This variant has been previously reported in 0.003% of HCM cases (O’Leary et al. 2016; Walsh et al. 2017; Nykamp et al. 2017). The alteration in the splicing site occurs at the border between an exon and an intron and can lead to the loss of exons or the inclusion of introns that also alter the protein sequence (Amberger et al. 2015).

The *TNNI3* gene (OMIM 191044) is located at 19q13.42 and codes for type 3 troponin I related to cardiac muscle contraction (Amberger et al. 2015; Walsh et al. 2017; Herrera-Rodriguez et al. 2020). On the other hand, *TPM1* (OMIM 191010) is located at 15q22.2 and encodes for tropomyosin 1. In the case of *TNN2* (OMIM 191045), it is located at 1q32.1 and encodes the cardiac isoform of troponin T type 2. These proteins are located in the thin filaments and regulate muscle contraction in response to changes in intracellular calcium ion concentration (O'Leary et al. 2016). Variants in these three genes have been associated with a family history of sudden death and other prognoses (Anan et al. 1998; Karibe et al. 2001; Rani et al. 2012; Renaudin et al. 2018).

The *SLC22A5* gene (OMIM 603377), located at 5q31.1, is a member of the organic cation transporter family and is expressed in the kidney, skeletal muscle, heart, and placenta (Amberger et al. 2015; Mutlu-Albayrak et al. 2015). Some variants in *SLC22A5* cause primary systemic carnitine deficiency, skeletal myopathy, or cardiomyopathy (O’Leary et al. 2016), due to a defect in the carnitine transporter. Patients present with hypoketotic hypoglycemia, HCM, and sudden death in children and adults (Frigeni et al. 2017). In our patient, the clinical history does not show any symptoms of primary carnitine deficiency.

An interesting case was found in family 6 where one of the members presented a deletion in *KCNE1* (OMIM 176261), located at 21q22.12 and belonging to the *KCNE* family of potassium channels (Chen, et al. 2003; Amberger et al. 2015). This gene codes for a transmembrane protein that, together with the *KVLQT1* gene product, forms the delayed rectifier potassium channel (Avalos Prado et al. 2021). There are few reports of deletions in *KCNE1* and those have been identified in patients with long QT syndrome (Splawski et al. 2000). Experimentally, a deletion in *KCNE1* has been found to increase susceptibility to atrial fibrillation in mice (Avalos Prado et al. 2021). This family member did not present symptoms of HCM but was included because his mother and brother were double heterozygotes for PV, in *MYH7* and *ACADVL*, which were not present in this patient.

The *ACADVL* gene (OMIM 609575) is located at 17p13.1 and codes for the very long chain of acyl-CoA dehydrogenase. Deficiency of this enzyme causes an inborn error in mitochondrial fatty acid β-oxidation that causes severe cardiomyopathy and/or sudden death during the neonatal period. This condition is rare and is inherited in an autosomal recessive manner. To our knowledge, there is only one report of a variant in *ACADVL* in a patient with HCM, caused by frameshift duplication (Kim et al. 2018). Due to the difference in PV present in this family, we suggest performing molecular studies in the other members, to confirm whether the PV in *KCNE1* is de novo and whether there are other heterozygotes for PV in *ACADVL* and *MYH7*.

Of the sporadic cases, a 7-year-old patient with severe symptoms of HCM stands out in whom only 2 VUS were found in *ACTC1* (OMIM 102540) and *EYA4* (OMIM 603550). The parents were negative for these VUS, which proves that they are de novo variants, and they do not report a history of HCM in the family. It is important to continue analyzing the presence of VUS to define its clinical importance in HCM since the meaning of these can change as more variants are categorized (Burke et al. 2022). It is suggested to perform a microarray study on the patient to see if these variants come from some de novo chromosomal rearrangement.

There is a great need to include the identification of gene variants to support the diagnosis of HCM, as the diagnosis is currently only made based on the patient’s symptoms and the results of imaging studies (Ommen et al. 2020). The identification of PV and PPV allows the detection of carriers in the family even before expressing symptoms of the disease. With this methodology, it is possible to distinguish double heterozygous patients, with two or more variants in different genes, where the clinical manifestations are more severe. Similarly, compound heterozygotes that present genetic variants in both alleles of the same gene, where the clinical phenotype leads to death in a few months (Rafael et al. 2017; Carrier 2021), could be identified. Currently, there are gene therapy proposals for compound heterozygotes for *MYBPC3* (Carrier 2021).

## Limitations

In this manuscript, we focus only on reporting the gene variants observed in our patients with HCM. To establish a prevalence of each of them in the Mexican population, it is necessary to increase the sample size of the population studied, as has been done in other populations (Erdmann et al. 2003; Richard et al. 2003; Van Driest et al. 2003; García-Castro et al. 2009; Otsuka et al. 2012; Saposnik et al. 2014).

## Informed Consent

Informed consent was obtained from all individual participants included in the study.

## Consent for publication

Consent for publication was obtained for every individual person’s data included in the study.

## Data Availability

The empirical data generated in this research is available upon request to the corresponding author, except for the personal data of the patients or any other data that could identify them.
