# Correction: Identification of variants in genes associated with hypertrophic cardiomyopathy in Mexican patients

**DOI:** 10.1007/s00438-023-02069-3

**Published:** 2023-10-20

**Authors:** Catalina García-Vielma, Luis Gerardo Lazalde-Córdova, José Cruz Arzola-Hernández, Erick Noel González-Aceves, Herminio López-Zertuche, Nancy Elena Guzmán-Delgado, Francisco González-Salazar

**Affiliations:** 1 Centro de Investigación Biomédica del Noreste, Departamento de Citogenética, Instituto Mexicano del Seguro Social, Monterrey, NL México; 2 Departamento de Electrofisiología, Instituto Mexicano del Seguro Social, Unidad Médica de Alta Especialidad. Hospital de cardiología No. 34 “Dr. Alfonso J. Treviño Treviño” del Centro Médico Nacional del Noreste, Monterrey, NL México; 3 Instituto Mexicano del Seguro Social, Hospital General de Zona No. 4, Monterrey, NL México

**Correction to: Correction to: Molecular Genetics and Genomics** 10.1007/s00438-023-02048-8

In this article, Nancy Elena Guzmán Delgado should have been denoted as a co-corresponding author.

The original article has been udpated.

